# Medical and social factors influencing the utilization of healthcare services among older adults in Israel during the COVID-19 lockdown

**DOI:** 10.3389/fpubh.2023.1218507

**Published:** 2023-09-27

**Authors:** Ohad Shaked, Liat Korn, Yair Shapiro, Moti Zwilling, Avi Zigdon

**Affiliations:** ^1^Department of Health Systems Management, School of Health Sciences, Ariel University, Ariel, Israel; ^2^Natali, Ramat Gan, Israel; ^3^Department of Economics and Business Administration, Ariel University, Ariel, Israel

**Keywords:** medical factors, medical service, older adults, COVID-19, social factor

## Abstract

**Background:**

The corona virus disease 2019 (COVID-19) pandemic significantly impacted older adults. However, most older communities focused on the medical issues. The aims of this study were to identify the medical and social factors linked with the usage of medical services during the COVID-19 lockdown in Israel.

**Methods:**

The study was conducted Over two periods of time from February to April in 2019 (P1), before the COVID-19 and from February to April in 2020 (P2), during the first lockdown. The study was conducted on people aged 65 and older in Israel. The variable statistics were analyzed using frequency tabulation, cross-tabulation frequencies, and t-tests. Two hierarchical logistic regressions were conducted over four steps for each period.

**Results:**

The participants (n = 102,303) comprised 64.5% female (65,946) and 35.5% male (36,357) (mean age 80.5, SD- 7.46). It was found that participants who had not subscribed to the supportive community services were 7.47 times more likely to access medical services in P1 and 12.417 times more likely to access medical services during the lockdown. This variable was also found to be a strong predictor in the final model. The most significant variable for predicting the participants’ needs during P2 was their previous needs in P1. Other social variables were living in assisted living home and living in community settlements. The presence of 12 diseases in this study did not predict service demand.

**Conclusion:**

Community support reduces medical service demands during disasters and provides services for older adults. During pandemics, however, social services need to be expanded and made more easily accessible to older adults.

## Introduction

During the first COVID-19 pandemic lockdown, health systems were often unprepared (1, 2). Because the COVID-19 pandemic more significantly affected older populations, the mortality rate for those aged 80 or older was 54% that is 5.1 times more than those who died aged 30–59 (3), 15% of the first wave of death were aged above 60, the mortality rate in age group 60–69 years was 3.6% (4). the Israeli government issued guidelines on isolation (5, 6) that instructed older adults to isolate in their homes to avoid exposure (5, 7). This meant that isolated older adults had to ask for assistance to purchase food and medicines from their immediate family or other people (2, 8).

Older adults with chronic medical conditions were more prone to catching COVID-19, had slower recoveries, and were more likely to have complications (9, 10). Therefore, it was vital that these people abided by the COVID-19 control measures, such as social distancing (11), to avoid the risk of more severe symptoms and hospitalization. COVID-19 was generally contracted through close contact with symptomatic and asymptomatic carriers, with the mildest symptoms reported by around 81% of infected people being coughs, sore throats, fever, muscle pain, and pulmonary embolism. However, severe cases reported pneumonia, shortness of breath, and low blood oxygen saturation, and 5% of infected people suffered from severe respiratory failure and septic shock (4, 12, 13).

Most severe adult COVID-19 cases had common comorbidities. For example, many hospitalized diabetics were at risk of dying from the COVID-19 virus, 75% of hospitalized patients were also suffering from hypertension, and cases with diseases such as chronic obstructive pulmonary disease (COPD), obesity, and cardiovascular diseases tended to have more serious infections that could lead to severe lung infections (11, 12, 14). The cardiovascular complications in many severe COVID-19 patients included acute myocardial infarction, myocardial infarction, myocarditis, heart failure, arrhythmia, and thrombosis (11, 14). Consequently, the mortality rates were significantly higher in people who had one or more chronic diseases (12, 14).

Even prior to the onset of the Covid-19 pandemic, numerous countries had already devised novel primary community care models aimed at addressing the medical needs of their communities. These models encompassed routine medical check-ups, the creation of medical records, exercise recommendations, and the establishment of centers catering to the needs of older individuals (assisted living home), medical lectures, and nursing and doctor home visits. However, these supportive communities were more focused on medical issues (15), such as ambulance services, home doctor visits, and telehealth advice. One such company providing these services in Israel was “Natalie Medical Services.”

This study was based on COVID-19 medical data from Natalie Medical Services in Israel during the lockdown period and medical data from the previous year, The company provides medical and logistical services to subscribers who pay a fixed fee each month and provided at the subscriber’s request (16). One of the services provided by Natalie Health Services is a supportive community service (16) that gives support, such as answering questions, giving advice, purchasing medicine and food, and other logistical or medical needs.

Therefore, this was a comprehensive longitudinal study based on data from the same Israeli population over two periods: before the start of the pandemic from February to April 2019 (P1), and during the government-ordered COVID-19 lockdown from February to April 2020 (P2). The purpose of this study was identifying the medical and social factors linked with the usage of.

medical services during the COVID-19 lockdown in Israel.

Previous studies found that high social involvement and belonging to a community support network were associated with higher well-being (17–20). Community support emphasizes the sharing of information about diseases, dealing with risk factors, counseling for complications, and information provision about available medical resources (21). Social isolation has been associated with less efficient disease management (22–24). Various underlying diseases, the most common of which are hypertension, cardiovascular diseases, and blood diseases, result in a greater need for ongoing medical services. As viruses can have more serious effects on chronically ill patients, such as people suffering from diabetics, hypertension, and respiratory illnesses (25–29).

## Materials and methods

The institutional ethical review committee of Ariel University gave ethical approval for this study (AU-HEA-AZ-20200624) after permission was given to access the data from *Natalie Medical Services in Israel*. This study was based on the longitudinal observations of older adults in two periods: before the COVID-19 pandemic from February to April 2019 (P1), and during the first lockdown in Israel from February to April 2020 (P2). The data for this study were anonymized using the company’s subscription number.

### Study population and sample

In the first period, the study population was *Natalie Medical Services in Israel* customers 65 and over, with the final study sample being participants who were presented in the data in both study periods.

After people who had canceled their subscriptions, passed away, had had their subscriptions frozen, or had missing data were removed, the sample comprised 102,303 participants (mean age- 80.5, SD- 7.46), and 64.5% (65946) of who were female and 35.5% (36357) of whom were male.

### Research tools

The study data collects customer information to monitor and supervise their needs and services. After anonymization, relevant variables from the Central Bureau of Statistics; residential socioeconomic status, sector, and religiosity; were added to the company subscription numbers (30).

### Variables

#### Dependent variable

The indexes for these four variables had Cronbach’s alpha scores at P1 of 0.693 and at P2 of 0.630. To construct the variable need index, the four variables: P1/P2 Emergency calls, P1/P2 Emergency Call Referrals, P1/P2 Ambulance, P1/P2 Doctor Visits were first recoded into dichotomous variables; 0- no service demand and 1- service demand; after which the variables were summed into one complex index for each period with a value range of 0–4 (see Table 1).

**Table 1:** Dependent variables description referring to two time periods P1/P2.

Variable	Description	P1 values*	P2 values*
P1/P2 Emergency Calls	Calls to the emergency call center for medical reasons	0–114	0–472
P1/P2 Emergency Call Referrals	Number of referrals for people who called the emergency call center	0–226	0–294
P1/P2 Ambulance	Number of times a customer ordered an ambulance	0–18	0–16
P1/P2 Doctor Visits	Number of times a doctor visited a client during these two periods	0–102	0–111

*Columns P1 and P2 represent pre COVID-19 period and during the lockdown period, Cronbach’s alpha for P1 = 0.693 and for P2 = 0.630.

#### Independent variables and sociodemographic variables

Table 2 presents the independent and variables and the description value of each variable.

**Table 2:** Independent variables description refers to residential configuration and background diseases.

Variable	Description	Category value
Supportive community services	Company service that ensured customers were contacted every week for assistance with non-medical needs, such as answering questions, counseling, purchasing food and medicines, and logistics	Yes- if this arrangement was supplied. No -if it was not supplied
Living in an assisted living home	If a customer lived in an assisted living home	Yes/No
Living in a community settlement	Determined based on the customer’s home address and matching with the Israeli Central Bureau of Statistics (CBS) (2019)	Yes/No
Housing type	If the address had a floor number, the housing type was considered a building, with all others being considered as private homes	Private house/ Apartment building
Cancer		0 = no disease, 1 = have disease
Blood diseases	Anemia, iron deficiencies, and low hemoglobin	
Hypertension		
Cardiovascular illnesses		
Neurological disorders	Cerebral vascular disease, Parkinson disease, and epilepsy	
Post-surgery issues		
Psychiatric problems		
Pulmonary diseases	COPD and asthma	
Diabetes		
Digestive issues		
Skeletal and mobility issues		
Kidney and urinary problems		

##### Sociodemographic variables

The sociodemographic variables were as follows: gender; age at the start date of the study in 2019 grouped into three categories (31); younger older adults (65 until 75), middle-aged older adults (75until 85), and older adults (85 and older); and family status divided into single, married, separated/ divorced, widowed, and not-reported. Of the sample 27,433 (26.8% of the sample) had missing family statuses. After cross-referencing with CBS data (2019), the additional sociodemographic variables derived from the respondent’s residential address were: settlement religiosity (1. secular, 2. religious, and 3. ultra-orthodox) and socioeconomic situations, which was first classified as per the CBS coding into clusters from 1 to 10, with 1 representing very low socioeconomic status and 10 representing very high socioeconomic status and then divided into two categories: 1. lower class- scores from 1 to 6; 2. high class- scores from 7 to 10.

### Data analysis

SPSS™ Statistics 27.0 software was used for the data analysis. The variable statistics descriptions were generated using frequency tabulation, cross-tabulation frequencies, and t-tests, the results for which are shown in Table 3. Hierarchical logistic regressions for each period were conducted, the results for which are shown in Table 4. Cross-tabulation frequencies and chi-square significance were used to compare the independent groups, the results for which are shown in Table 5.

**Table 3:** Variables and indexes socio-demographics, social, disease, and needs for services.

	Variables	Values	%	*n*
Socio-demographics	Gender	Female	64.5	65,946
		Male	35.5	36,357
	Age	65–119		
	Family status	Single	1.1	1,101
		Married	45.0	46,063
		Separated/ Divorced	4.4	4,491
		Widowed	22.7	23,215
		Not reported	26.8	27,433
	Socioeconomic status (SES) Clusters 1–10 dichotomized by the median	Low SES (1–6)	54.9	56,135
		High SES (7–10)	45.1	46,168
Social	Living in a community settlement	Yes	2.9	2,981
		No	97.1	99,322
	Living in an assisted living home	Yes	26.4	26,963
		No	73.6	75,340
	Supportive community services	0 = Subscribed	2.2	2,283
		1 = Not subscribed	97.8	100,020
	Living in apartment building	Yes	80.1	79,908
		No	19.9	19,911
Disease	Diabetes	0 = no disease, 1 = disease	17.2	17,597
	Lung problems		8.7	8,903
	Psychiatric issues		2.6	2,699
	Post-surgery issues		5.3	5,440
	Neurological issues		9.1	9,265
	Cardiovascular issues		23.8	24,301
	Hypertension		41.0	41,902
	Blood		22.7	23,254
	Cancer		5.1	5,188
	Digestive issues		4.7	4,759
	Skeletal issues		5.8	5,891
	Disease index	Scale 0–11		
Needs for services in P1 and P2 scale	P1 needs and P2 needs: Medical calls (0,1), Emergency calls (0,1), Ambulance orders (0,1), Doctors home visits (0,1)	0 needs in P1	66.9	68,441
		1 or more needs in P1	33.1	33,862
		0 needs in P2	76.0	77,768
		1 or more needs in P2	24.0	24,535

SES= Socioeconomic status.

**Table 4:** 4 steps Hierarchical logistic regressions for needs predictions in both periods for the study variables.

	Measures	Values	Prediction of needs in P1 before COVID-19			Prediction of the needs in P2 during COVID-19 lockdown				
			Exp(B)/RR	CI		Explained var	Exp(B)/RR	CI		Exp var
Step 1	Gender Age Family status SES	1 = Male, 2 = Female From age 65 to 119 0 = marital, 1 = Not marital 0 = Higher, 1 = Lower	0.946*** 1.437*** 2.073*** 1.115***	0.884 1.375 1.938 1.048	0.914 1.406 2.004 1.081	6.2%	0.984** 1.542*** 1.943*** 1.155***	0.912 1.470 1.806 1.079	0.947 1.506 1.873 1.116	6.0%
Step 2	Gender	1 = Male, 2 = Female	0.898***	0.868	0.930	**9.3%**	0.928***	0.893	0.964	**9.6%**
	Age	From age 65 to 119	1.268***	1.239	1.298		1.335***	1.301	1.369	
	Family status	0 = marital, 1 = Not marital	1.789***	1.725	1.855		1.645***	1.581	1.711	
	SES	0 = Higher, 1 = Lower	0.972	0.941	1.003		0.994	0.960	1.030	
	Supportive community service	0 = Belong, 1 = Not belong	5.761***	4.413	7.520		9.187***	6.054	13.943	
	Living in assisted living home	0 = No, 1 = Yes	1.926***	1.860	1.995		2.070***	1.993	2.149	
	Living in community settlements	0 = No, 1 = Yes	1.088	0.977	1.211		1.555***	1.363	1.775	
	Living alone	0 = No, 1 = Yes	1.152***	1.099	1.208		1.134***	1.078	1.194	
	Living in apartment building	0 = No, 1 = Yes	1.049*	1.004	1.096		1.110***	1.057	1.165	
Step 3	Gender	1 = Male, 2 = Female	0.934***	0.902	0.968	**13.4%**	0.972	0.935	1.011	**15.1%**
	Age	From age 65 to 119	1.202***	1.174	1.231		1.255***	1.223	1.289	
	Family status	0 = marital, 1 = Not marital	1.809***	1.743	1.877		1.670***	1.603	1.739	
	Supportive community service	0 = Subscribed, 1 = Not subscribed	7.472***	5.721	9.760		12.417***	8.164	18.884	
	Living in older adults homes	0 = No, 1 = Yes	2.148***	2.072	2.226		2.402***	2.311	2.497	
	Living in community settlements	0 = No, 1 = Yes	**–**	**–**	**–**		1.510***	1.321	1.726	
	Living alone	0 = No, 1 = Yes	1.138***	1.086	1.193		1.099***	1.045	1.155	
	Living in apartment building	0 = No, 1 = Yes	1.052*	1.008	1.097		1.108***	1.054	1.165	
	Disease	0 = No, 1 = Yes	1.231***	1.221	1.241		1.281***	1.270	1.292	
Step 4	Age	From age 65 to 119					1.188***	1.154	1.224	**37.9%**
	Family status	0 = marital, 1 = Not marital					1.312***	1.255	1.370	
	Supportive community service	0 = Belong, 1 = Not belong					6.449***	4.189	9.930	
	Living in assisted living home	0 = No, 1 = Yes					1.871***	1.790	1.955	
	Living in community settlements	0 = No, 1 = Yes					1.649***	1.422	1.913	
	Living alone	0 = No, 1 = Yes					1.040	0.983	1.100	
	Living in apartment building	0 = No, 1 = Yes					1.107***	1.047	1.171	
	Disease	0 = No, 1 = Yes					1.205***	1.193	1.217	
	P1 needs	0 = No, 1 = Yes					10.002***	9.605	10.416	

*p < 0.05, **p < 0.01, ***p < 0.001; SES, Socioeconomic status; Exp(B), Value of adjusted relative risk; CI, Confidence interval: Explained Variance, value of Nagelkerke *R* square.

**Table 5:** Comparison of study variables by those subscribed to the supportive community service versus the needs.

Variables	Values	Subscribed to SCS	Unsubscribed to SCS	*Sig.	No needs in P1	Needs in P1	*Sig.
Gender	Female	**71.4%**	64.3%	***	63.5%	**66.3%**	***
	Male	28.6%	35.7%		36.5%	33.7%	
Age groups	Younger	7.0%	29.6%	***	32.7%	21.7%	***
	Middle-aged	40.6%	46.2%		45.3%	**47.5%**	
	Older	**52.4%**	24.3%		22.0%	30.8%	
Family status	marital	**72.6%**	61.5%	***	67.8%	49.5%	***
	Not marital	27.4%	38.5%		32.2%	**50.5%**	
SES cluster	Low	33.9	45.4%	***	44.2%	**47.0%**	***
	High	**66.1%**	54.6%		55.8%	53.0%	
Community settlement	Yes	**24.7%**	2.4%	***	3.2%	2.4%	***
	No	75.3%	97.6%		96.8%	**97.6%**	
Buildings	Private house	15.5%	20.0%	***	20.9%	18.1%	***
	Apartment building	**84.5%**	80.0%		79.1%	**81.9%**	
Disease index	No disease	32.1%	46.5%	***	52.1%	34.1%	***
	One or more disease	**67.9%**	53.5%		47.9%	**65.9%**	
P1 needs	No needs	**95.1%**	66.3%	***			
	One need or more	4.9%	33.7%				
Supportive community services	Subscribed to SCS				3.2%	0.3%	***
	Unsubscribed to SCS				96.8%	**99.7%**	

Chi-square significance for differences between the independent groups. Modes are bolded. ***p < 0.001; SCS=Supportive community service.

## Results

Table 3 presents the variables and the indexes used in the regression model and the percentages for each value.

Table 4 shows the results for the hierarchical logistic regressions that predicted the needs before COVID-19 (P1) and during the first COVID-19 lockdown in Israel (P2). The analyses were conducted over four steps: 1. Sociodemographic variables as gender, age, family status, socioeconomic status (SES); 2. The sociodemographic variables were added to the social indices such as living in a communal settlement, living in an apartment building with supportive community services and in apartment buildings, living in assisted living homes, living in assisted living homes, suggesting that the older are not living in loneliness, social isolation, and they have limited social support (32). Data from the first step were entered, and the member variables were added; 3. the significant variables from the previous steps and the disease index were entered; and 4. the needs in the previous period (P1) were entered. The final model predicting the extent of the needs during the COVID-19 lockdown was relevant only to P2 and explained 38% of the variance.

In step 3 of both periods, customers who were not subscribed to the supportive community services (RR = 7.472, *p* < 0.001, CI 5.721, 9.760 in P1 and RR = 12.417, *p* < 0.001, CI 8.164, 18.884 in P2) were 7.472 times more likely to require medical services in P1 and 12.417 times more likely to require medical services during the COVID-19 lockdown. This variable continued to be a strong predictor in the final model (RR = 6.449, *p* < 0.001, CI 4.189, 9.930).

The final P2 analysis model found that the most significant variable for needs prediction during P2 was the previous needs in P1 (RR = 10.002, *p* < 0.001, CI 9.605, 10.416). Another social variable was living in an assisted living home (RR = 1.871, *p* < 0.001, CI 1.790, 1.995) and living in a community settlement (RR = 1.649, *p* < 0.001, CI 1.422, 1.913).

To understand the nature of these strong prediction variables on the need’s requirements in P2, a comparison was conducted between the independent groups subscribed to the supportive community services in P1 (Table 5). It appears that there were significant differences between the groups for all study variables. Compared with people who chose not to subscribe to the supportive community service (SCS), more subscribers were: female, from the older group, married, from a high SES, living in a community settlement, living in apartment buildings, and had had no needs in the previous year. Higher P1 service need frequencies were female, from the middle-aged group, single, from a low SES, not living in community settlements, living in apartment building s, had one or more diseases, and was unsubscribed to the SCS. The diseases recorded in the subscriber’s medical records were diabetes, respiratory diseases, mental health diseases, post-surgery needs, neurological diseases, cardiovascular diseases, hypertension, blood diseases, cancer, intestinal diseases, skeletal and muscular diseases, and urinary system diseases. The social characteristics of the subscribers were: family status, living in community settlements, living in assisted living home, and belonging to a supportive community. Assisted living homes are designed for older adults who are independent and need less nursing help; they also empower the information of health, treatments, and health services. Older adults tend to use less information than younger adults, showing that the awareness of older adults about their rights is less compared to nursing people who need a close medical environment, as in a nursing home (33, 34).

## Discussion

A supportive community environment creates a lower demand for medical services and is necessary for situations when older adults need assistance (35–37). This study examined factors affecting the demand for medical services by older adults with different medical and social characteristics in Israel during the COVID-19 lockdown. This study was based on a large adult sample of 103,955 members of Natalie Health Services in both periods. It was hypothesized that the medical needs of patients who received social support would be lower during the first quarantine period.

The results from the two research periods showed that during the first COVID-19 lockdown period, the referral frequencies of older adults to health service providers for various social and medical issues were significantly lower compared with the corresponding period in 2019. As expected, the social factors like living in community settlement, and having a supportive community service reduced the need for services by 0.8 in P2, as was also observed in previous literature (38–40). This suggests that the availability of a supportive community environment and services played a role in reducing the need for medical services during the lockdown.

Supportive community service (16, 41) gives support, such as answering questions, giving advice, purchasing medicine and food, and other logistical or medical needs. The study revealed that individuals affiliated with supportive community services required fewer medical interventions in both time periods. This not only lessened the chances of complications but also lowered the demand for medical services. Furthermore, during the second period (P2), the need for medical care was notably reduced among individuals with chronic illnesses. This decline could be attributed to their access to social services and support, potentially leading them to forgo seeking medical attention (42–46).

It is recommended that services like supportive community services should be provided to chronically ill people and social programs prioritized for people with more complex conditions to reduce the risk of complications and decrease the need for medical services potentially because they had access to social services and other forms of support. The significant differences between the groups suggested that the variances between these groups could be explain by the strong effects of supportive community services. To better serve vulnerable populations, governments and governmental organizations should enhance their engagement by allocating larger budgets for social programs. This step involves both strengthening current initiatives that support older individuals and chronic patients and fortifying these programs. The objective is to bolster their capacity to aid during emergencies, consequently mitigating the strain on medical services. Ultimately, reinforcing existing programs aimed at assisting the older adults and chronic patients not only diminishes the demand for medical services during crises but also guarantees essential support for these susceptible demographics.

## Conclusion

During the first quarantine period, the medical service consumption was lower. Chronic patients, especially those with hypertension, cardiovascular diseases, and polypharmacy, utilized medical services frequently. During medical crises, such as the COVID-19 pandemic, social services must be expanded and made more accessible to older adults. Organizations that provide medical services to chronic patients, such as health maintenance organization, HMO and national insurance services, should activate and support social programs for complex patients to reduce the risk of complications and consumption of services. Supportive communities have a dual impact by decreasing the need for medical services during emergencies and providing vital assistance to older individuals. To achieve this, governments and government organizations should develop programs that boost social engagement, focusing on supporting older and chronically ill individuals in times of crisis. By implementing measures like fostering supportive community environments and offering services such as supportive community services, the pressure on medical services can be significantly alleviated, particularly during critical situations. Prioritizing social programs and support for vulnerable populations, such as older individuals and chronically ill patients, is crucial to ensuring their well-being and reducing the strain on medical services during challenging situations like a pandemic.

### Strengths and limitations

This study was based on a large group of older adults in Israel and was intended to prospectively evaluate the risk and protective factors related to their medical needs. The research design and sample were advantages that gave strong support to the study conclusions. However, as this was an observational study, the causal relations between the variables could not be confirmed because of the possible interference of confounder variables. Another limitation may stem from the medical conditions of those living in the assisted living home compared with those living in their own homes as the people living in assisted living home may have had more severe medical conditions. Another limitation arose due to the uncertainty surrounding the variable of assisted living homes. This uncertainty was because no significant differences were observed between individuals living in assisted living homes and those residing in nursing homes.

## Data availability statement

The data analyzed in this study is subject to the following licenses/restrictions: Data cannot be shared publicly as it is confidential to Natali privet company. The first author (OS) is the chief manager of “Natali Healthcare Solutions” call center. Natali’s CEO gave had permission to use Natali’s database for this study. The database was collected as part of services provided by “Natali Healthcare Solutions” to its customers in Israel. The data underlying the results presented in the study are available from “Natali Healthcare Solutions.” The company URL: http://Natali.co.il. The authors do not have permission to share the data. OS: ohads@natali.co.il. Requests to access these datasets should be directed to OS, Natali Healthcare Solutions, ohads@natali.co.il.

## Ethics statement

The studies involving humans were approved by Ariel university AU-HEA-AZ-20200624. The studies were conducted in accordance with the local legislation and institutional requirements. Written informed consent for participation was not required from the participants or the participants’ legal guardians/next of kin in accordance with the national legislation and institutional requirements.

## Author contributions

OS, AZ, and LK: investigation. OS: original draft preparation and data curation. LK and OS: formal analysis. OS, AZ, LK, YS, and MZ: conceptualization, review, and editing. AZ and YS: supervision. All authors have read and agreed to the published version of the manuscript.

## References

[1] Omboni S. Telemedicine during the COVID-19 in Italy: a missed opportunity? Telemed J E Health. (2020) 26:973–5. doi: 10.1089/tmj.2020.0106 32324109 PMC7415870

[2] Rai P Kumar BK Deekshit VK Karunasagar I Karunasagar I. Detection technologies and recent developments in the diagnosis of COVID-19 infection. Appl Microbiol Biotechnol. (2021) 105:441–5. doi: 10.1007/s00253-020-11061-5 33394144 PMC7780074

[3] Zhang J-J Dong X Liu G-H Gao Y-D. Risk and protective factors for COVID-19 morbidity, severity, and mortality. Clin Rev Allergy Immunol. (2023) 64:90–7. doi: 10.1007/s12016-022-08921-5 35044620 PMC8767775

[4] Banerjee D. The impact of Covid-19 pandemic on elderly mental health. Int J Geriatr Psychiatry. (2020) 35:1466–7. doi: 10.1002/gps.5320 32364283 PMC7267435

[5] Wirth R Becker C Djukic M Drebenstedt C Heppner HJ Jacobs AH. COVID-19 in old age-the geriatric perspective. Z Gerontol Geriatr. (2021) 54:152–09. doi: 10.1007/s00391-021-01864-0 33595696 PMC7887547

[6] Hossain MA Jahid MIK Hossain KMA Walton LM Uddin Z Haque MO. Knowledge, attitudes, and fear of COVID-19 during the rapid rise period in Bangladesh. PLoS One. (2020) 15:e0239646. doi: 10.1371/journal.pone.02396464 32970769 PMC7514023

[7] Brooke J Jackson D. Older people and COVID-19: isolation, risk and ageism. J Clin Nurs. (2020) 29:2044–6. doi: 10.1111/jocn.152745 32239784

[8] Kakodkar P Kaka N Baig MN. A comprehensive literature review on the clinical presentation, and management of the pandemic coronavirus disease 2019 (COVID-19). Cureus. (2020) 12:e7560. doi: 10.7759/cureus.7560 32269893 PMC7138423

[9] Long B Brady WJ Koyfman A Gottlieb M. Cardiovascular complications in COVID-19. Am J Emerg Med. (2020) 38:1504–7. doi: 10.1016/j.ajem.2020.04.048 32317203 PMC7165109

[10] Marti CN Georgiopoulou VV Giamouzis G Cole RT Deka A Tang WHW. Patient-reported selective adherence to heart failure self-care recommendations: a prospective cohort study: the Atlanta cardiomyopathy consortium: selective self care adherence in heart failure. Congest Heart Fail. (2013) 19:16–24. doi: 10.1111/j.1751-7133.2012.00308.x 22958604 PMC3519965

[11] Jankowska-Polańska B Świątoniowska-Lonc N Sławuta A Krówczyńska D Dudek K Mazur G. Patient-reported compliance in older age patients with chronic heart failure. PLoS One. (2020) 15:e0231076. doi: 10.1371/journal.pone.0231076 32298283 PMC7161980

[12] Halpin DMG Criner GJ Papi A Singh D Anzueto A Martinez FJ. Global initiative for the diagnosis, management, and prevention of chronic obstructive lung disease. The 2020 GOLD science committee report on COVID-19 and chronic obstructive pulmonary disease. Am J Respir Crit Care Med. (2021) 203:24–36. doi: 10.1164/rccm.202009-3533SO 33146552 PMC7781116

[13] Gasmi A Peana M Pivina L Srinath S Gasmi Benahmed A Semenova Y. Interrelations between COVID-19 and other disorders. Clin Immunol. (2021) 224:108651. doi: 10.1016/j.clim.2020.108651 33333255 PMC7833539

[14] Ochani R Asad A Yasmin F Shaikh S Khalid H Batra S. COVID-19 pandemic: from origins to outcomes. A comprehensive review of viral pathogenesis, clinical manifestations, diagnostic evaluation, and management. Infez Med. (2021) 29:20–36. Available at: https://infezmed.it/media/journal/Vol_29_1_2021_3.pdf 33664170

[15] Li Q Zhao C. A review of the current status of clinical management of COVID-19 in the elderly. Med Sci Monit. (2021) 27:e930278. doi: 10.12659/MSM.930278 33833211 PMC8043417

[16] Gu T Yuan J Li L Shao Q Zheng C. Demand for community-based care services and its influencing factors among the elderly in affordable housing communities: a case study in Nanjing City. BMC Health Serv Res. (2020) 20:241. doi: 10.1186/s12913-020-5067-0 32293427 PMC7092588

[17] Natali Company. (2023). Available at: https://www.natali.co.il/en/about-natali/ (Accessed April 1, 2023).

[18] Mohamadi M Goodarzi A Aryannejad A Fattahi N Alizadeh-Khoei M Miri S. Geriatric challenges in the new coronavirus disease-19 (COVID-19) pandemic: a systematic review. Med J Islam Repub Iran. (2020) 34:123. doi: 10.47176/mjiri.34.123 33437719 PMC7787036

[19] Singu S Acharya A Challagundla K Byrareddy SN. Impact of social determinants of health on the emerging COVID-19 pandemic in the United States. Front Public Health. (2020) 8:406. doi: 10.3389/fpubh.2020.00406 32793544 PMC7385373

[20] Shapiro O Gannot RN Green G Zigdon A Zwilling M Giladi A. Risk behaviors, family support, and emotional health among adolescents during the COVID-19 pandemic in Israel. Int J Environ Res Public Health. (2022) 19:3850. doi: 10.3390/ijerph19073850 35409535 PMC8997377

[21] Halevi Hochwald I Radomyslsky Z Danon Y Nissanholtz-Gannot R. Challenges in home care at the end stage of dementia: hospice units vs. home care units. A qualitative study. Death Stud. (2022) 46:1667–77. doi: 10.1080/07481187.2020.1829748 33040716

[22] Chaix B Isacsson S-O Råstam L Lindström M Merlo J. Income change at retirement, neighbourhood-based social support, and ischaemic heart disease: results from the prospective cohort study “men born in 1914.”. Soc Sci Med. (2007) 64:818–9. doi: 10.1016/j.socscimed.2006.10.018 17126973

[23] Liao J Wu X Wang C Xiao X Cai Y Wu M. Couple-based collaborative management model of type 2 diabetes mellitus for community-dwelling older adults in China: protocol for a hybrid type 1 randomized controlled trial. BMC Geriatr. (2020) 20:123. doi: 10.1186/s12877-020-01528-5 32228462 PMC7106607

[24] Martire LM Helgeson VS. Close relationships and the management of chronic illness: associations and interventions. Am Psychol. (2017) 72:601–2. doi: 10.1037/amp0000066 28880106 PMC5598776

[25] Park JM Sohn A. Predictors affecting the elderly’s use of emergency medical services. Osong Public Health Res Perspect. (2020) 11:209–5. doi: 10.24171/j.phrp.2020.11.4.10 32864312 PMC7442443

[26] Panagiotou OA Kosar CM White EM Bantis LE Yang X Santostefano CM. Risk factors associated with all-cause 30-day mortality in nursing home residents with COVID-19. JAMA Intern Med. (2021) 181:439–48. doi: 10.1001/jamainternmed.2020.7968 33394006 PMC7783593

[27] Næss G Kirkevold M Hammer W Straand J Wyller TB. Nursing care needs and services utilised by home-dwelling elderly with complex health problems: observational study. BMC Health Serv Res. (2017) 17:645. doi: 10.1186/s12913-017-2600-x 28899369 PMC5596938

[28] Zhou Y Chi J Lv W Wang Y. Obesity and diabetes as high-risk factors for severe coronavirus disease 2019 (Covid-19). Diabetes Metab Res Rev. (2021) 37:e3377. doi: 10.1002/dmrr.3377 32588943 PMC7361201

[29] Peric S Stulnig TM. Diabetes and COVID-19: disease-management-people. Wien Klin Wochenschr. (2020) 132:356–1. doi: 10.1007/s00508-020-01672-3 32435867 PMC7238399

[30] Grasselli G Greco M Zanella A Albano G Antonelli M Bellani G. Risk factors associated with mortality among patients with COVID-19 in intensive care units in Lombardy, Italy. JAMA Intern Med. (2020) 180:1345–55. doi: 10.1001/jamainternmed.2020.3539 32667669 PMC7364371

[31] Israeli Central Bureau of Statistics (CBS). (2023). Available at: https://www.cbs.gov.il/EN/settlements/Pages/default.aspx?mode=Yeshuv (Accessed April 13, 2023).

[32] Wilson-Genderson M Heid AR Cartwright F Collins AL Pruchno R. Change in loneliness experienced by older men and women living alone and with others at the onset of the COVID-19 pandemic. Res Aging. (2022) 44:369–81. doi: 10.1177/01640275211026649 34344251 PMC9039590

[33] Hughes CM Lapane KL Mor V Turrell A Castleden CM. Impact of legislation on nursing home care in the United States: lessons for the united Kingdom Commentary: a new script for nursing home care in the United Kingdom? BMJ. (1999) 7216:1060–3. doi: 10.1136/bmj.319.7216.1060 PMC1116850 10521206

[34] Roth-Cohen O Levy S Zigdon A. The mediated role of credibility on information sources and patient awareness toward patient rights. Int J Environ Res Public Health. (2021) 18:8628. doi: 10.3390/ijerph18168628 34444377 PMC8392652

[35] Ryu SI Cho B Chang SJ Ko H Yi YM Noh E-Y. Factors related to self-confidence to live alone in community-dwelling older adults: a cross-sectional study. BMC Geriatr. (2021) 21:291. doi: 10.1186/s12877-021-02214-w 33947334 PMC8097788

[36] Shaked O Korn L Shapiro Y Koren G Zigdon A. Socio-demographic characteristics and their relation to medical service consumption among elderly in Israel during the COVID-19 lockdown in 2020 as compared to the corresponding period in 2019. PLoS One. (2022) 17:e0278893. doi: 10.1371/journal.pone.0278893 36520880 PMC9754223

[37] Shaked O Korn L Shapiro Y Zigdon A. Social factors contributing to healthcare service requirements during the first COVID-19 lockdown among older adults. Healthcare (Basel). (2022) 10:1854. doi: 10.3390/healthcare10101854 36292300 PMC9601430

[38] Yang L Wang L Di X Dai X. Utilisation of community care services and self-rated health among elderly population in China: a survey-based analysis with propensity score matching method. BMC Public Health. (2021) 21:1936. doi: 10.1186/s12889-021-11989-x 34696767 PMC8546940

[39] Hartnett KP Kite-Powell A De Vies J Coletta MA Boehmer TK Adjemian J. Impact of the COVID-19 pandemic on emergency department visits - United States, January 1, 2019-May 30. MMWR Morb Mortal Wkly Rep (2020). (2020) 69:699–4. doi: 10.15585/mmwr.mm6923e1 PMC7315789 32525856

[40] Masroor S. Collateral damage of COVID-19 pandemic: delayed medical care. J Card Surg. (2020) 35:1345–7. doi: 10.1111/jocs.14638 32419177 PMC7276840

[41] David J Sibikova M Amaratunga SA Lebl J. COVID-19 pandemic in the Czech Republic: substantial decline of the demand for pediatric healthcare services. Klin Padiatr. (2021) 233:40–2. doi: 10.1055/a-1268-9211 33105512

[42] Ministry of welfare-division of social services. Instruction provisions for elderly supporting community 2022 (2020). Available at: https://clickrevaha.molsa.gov.il/product-page/610 (accessed April 18, 2023).

[43] Müller F Hummers E Jablonka A Schmidt T Noack EM. Auswirkung des COVID-19-Lockdowns auf Rettungseinsätze. Notf Rett Med. (2022) 25:341–7. doi: 10.1007/s10049-021-00873-1 33903799 PMC8060906

[44] Slagman A Behringer W Greiner F Klein M Weismann D Erdmann B. AKTIN emergency department registry, German forum of university emergency departments (FUN) in the Society of University Clinics of Germany E.V. medical emergencies during the COVID-19 pandemic. Dtsch Arztebl Int. (2020) 117:545–2. doi: 10.3238/arztebl.2020.0545 32865489 PMC8171546

[45] Tschaikowsky T Becker von Rose A Consalvo S Pflüger P Barthel P Spinner CD. Numbers of emergency room patients during the COVID-19 pandemic. Notf Rett Med. (2021) 24:943–2. doi: 10.1007/s10049-020-00757-w 32837303 PMC7341467

[46] Barten DG Latten GHP van Osch FHM. Reduced emergency department utilization during the early phase of the COVID-19 pandemic: viral fear or lockdown effect? Disaster Med Public Health Prep. (2022) 16:36–9. doi: 10.1017/dmp.2020.303, PMID: 32782063 PMC7503047

